# Predictive value of random blood glucose versus fasting blood glucose on in-hospital adverse events in patients with ST-segment elevation acute myocardial infarction

**DOI:** 10.1186/s12872-020-01394-4

**Published:** 2020-02-27

**Authors:** Yuhan Qin, Gaoliang Yan, Yong Qiao, Dong Wang, Erfei Luo, Jiantong Hou, Chengchun Tang

**Affiliations:** 1 Medical school of Southeast University, Nanjing, 210009 People’s Republic of China; 2 Department of Cardiology, Zhongda Hospital affiliated to Southeast University, Nanjing, 210009 People’s Republic of China

**Keywords:** RBG, FBG, STEMI, Adverse events, Prognosis

## Abstract

**Background:**

We aim to find out the relationship between random blood glucose (RBG), fasting blood glucose (FBG) and in-hospital adverse events in ST-segment elevation acute myocardial infarction (STEMI) patients. We evaluate and compare the predictive value of RBG and FBG on in-hospital adverse events, and give an appropriate cut-off value of RBG and FBG.

**Method:**

A retrospective study enrolled 958 consecutive AMI patients undergoing emergency coronary angiography at Zhongda Hospital were enrolled from January 1, 2016, to December 31, 2018 was performed. RBG and FBG, baseline data and adverse events were recorded. Major adverse cardiovascular and cerebrovascular events (MACCE) were defined as death, nonfatal recurrent myocardial infarction and stroke. Other adverse events included malignant arrhythmia, cardiac shock and hemorrhage. Patients with RBG > 11.1 mmol/L were divided into elevated RBG group. Patients with FBG > 6.1 mmol/L were divided into elevated FBG group. The incidence of in-hospital adverse events were compared in elevated RBG/FBG group and the control group. ROC curve was used to evaluate the predictive value of RBG and FBG on in-hospital adverse events.

**Result:**

The incidence of death, hemorrhage, cardiac shock and malignant arrhythmia significantly increases in elevated RBG and FBG group. Binary logistic regression showed that age, hypertension, diabetes, FBG and RBG were independent risk factors for in-hospital adverse events in STEMI patients. The AUC and 95% CI of RBG and FBG in predicting death of AMI patients were 0.789, 0.759~0.816; 0.810, 0.783~0.835, respectively. The cut-off values ​were 13.82 and 7.35 mmol/L. RBG and FBG also had fine predictive value on cardiac shock and malignant arrhythmia, no statistical difference was found in the predictive value on in-hospital adverse events (*P* = 0.462, *P* = 0.570, *P* = 0.694).

**Conclusion:**

Incidence of in-hospital adverse events significantly increases in AMI patients combined with elevated RBG or FBG. Both RBG and FBG were independent risk factors for in-hospital adverse events, they had good value on predicting in-hospital adverse events and there was no statistical difference in their predictive value.

## Background

In modern countries, mortality after AMI has been significantly decreased with coronary revascularization developments [1], nevertheless, AMI still plays an important role in cardiovascular disease, and account for a large proportion in the causes of human death. Diabetes mellitus is becoming a burden on society because of its endemic increase and high cardiovascular morbidity and mortality [2]. The morbidity and mortality of cardiovascular disease in diabetic patients is 2–3 times higher than that in normal glucose population [3]. Macrovascular complications such as myocardial infarction and stroke account for 80% of all deaths in patients with T2DM [4]. Moreover, elevated blood glucose is common in patients with AMI.

Epidemiological studies have demonstrated that hyperglycemia is an independent risk factor for poor prognosis in AMI patients, whether or not having diabetes history [5]. The prognostic value of the blood glucose in AMI patients was first suggested in 1975 [6], numerous studies have researched the correlation between hyperglycemia and adverse outcome in AMI patients since then. Hyperglycemia on admission is associated with higher mortality incidence, larger infarct size, impaired left ventricular function and poor clinical prognosis after AMI [5], Researches demonstrated a linear correlation between blood glucose on admission and AMI mortality. Mikhail Kosiborod and his colleagues demonstrated that persistent hyperglycemia during AMI is more predictive of mortality than admission blood glucose [7]. Among non-diabetic patients with AMI, elevated admission blood glucose is also proved to be associated to more severe multivessel coronary disease and deteriorates the short-term prognosis [8]. Furthermore, prediction models for long-term mortality in AMI patients were developed for risk stratification, closer follow-up and secondary prevention are necessary in high-risk patients [9].

Elevated fasting glucose is common in patients with acute myocardial infarction (AMI) and is associated with an increased short-term mortality [10]. It is reported IFG as well as IGT is an independent predictor of all-cause mortality in AMI patients [11]. A Korean prospective study including more than 1 million participants revealed that fasting glucose between 6.1–6.9 mmol/L was significantly related to increased risk of AMI [12], in the 4 year-follow-up Framingham Heart Study enrolled 4138 participants, IFG was associated with a higher cardiovascular disease risk in women but not in men [13]. There is no clear cut-off value of blood glucose to predict in-hospital poor prognosis. In this research, we aim to evaluate and compare the predictive value of RBG and FBG on in-hospital adverse events and provide an appropriate cut-off value for RBG and FBG to predict in-hospital adverse events.

## Methods

### Study population

A total of 958 consecutive patients with AMI underwent emergency coronary angiography in the Department of Cardiology, Zhongda Hospital affiliated to Southeast University, were enrolled from January 1, 2016 to December 31, 2018. The enrollment criteria were: 1. Patients with AMI underwent emergency coronary angiography; 2. Aged 18–80; exclusion criteria were as followed: 1. People who were allergic to iodine or iodine contrast agents; 2. Patients with intensive hemodynamic instability, severe liver or kidney dysfunction, severe infectious diseases, advanced malignant tumors, severe hematological diseases, or incomplete clinical data; all patients signed informed consent before enrollment.

### Study methods

Baseline data including demographic data, blood pressure, Killip grade, anamnesis and personal history were all recorded. Standard methods were used to measure the resting systolic blood pressure, diastolic blood pressure and BMI [14]. The Hematological examination indicators including plasma blood glucose were measured in the laboratory of our hospital. Echocardiography parameters, medications and coronary angiography data were also recorded. Further information can be found in previous publication [15].

In-hospital adverse events were defined as: major adverse cardiovascular and cerebrovascular events (MACCE) including death, recurrent nonfatal myocardial infarction, and other in-hospital adverse events including hemorrhage, cardiogenic shock and malignant arrhythmia. After effective coronary intervention therapy, chest pain and TNI significantly improved. Recurrent nonfatal myocardial infarction is defined as chest pain appeared again, TNI increased again during hospitalization, and coronary angiography confirmed the recurrence of AMI. Hemorrhage referred to life-threatening hemorrhage associated with the use of antiplatelet and anticoagulant drugs such as massive gastrointestinal hemorrhage and intracranial hemorrhage.

Hyperglycemia: The American Diabetes Association defines hospital related hyperglycemia as FBG > 6.9 mmol/L or RBG > 11.1 mmol/L without diabetes history, diabetic patients who are always above the normal blood glucose upper limit could also be classified into hyperglycemia group (14). In the research, RBG > 11.1 mmol/L was defined as elevated RBG regardless of a history of diabetes. In addition, FBG > 6.1 mmol/L was defined as elevated FBG. Patients admitted with RBG < 11.1 mmol/L were included in the control RBG group and patients with FBG < 6.1 mmol/L were included in the control FBG group. Venous blood was collected and RBG was tested as soon as the patients were admitted to the hospital before starting treatments such as revascularization, FBG was detected by collecting venous blood on the next morning after admission to the hospital. RBG and FBG were all measured in all patients.

Malignant arrythmia: Ventricular fibrillation, ventricular tachycardia, polymorphic Ventricular pre-contraction, paroxysmal supraventricular tachycardia.

Smoking: continuous or cumulative smoking for at least 6 months in a lifetime.

ST-segment elevation myocardial infarction referred to the STEMI criteria according to the fourth universal definition of myocardial infarction [16].

### Statistical analysis

SPSS 19.0 and MedCalc v18.11.3 software were used for statistical analyses. Categorial data was expressed as cases and percentages, and the χ2 test was used for analysis. Continuous data was expressed as mean ± Standard deviation, and compared using the independent samples t-test. Non-normally distributed numerical data were expressed as median and 25th–75th interquartile range, and compared using a rank-sum test. Univariate and binary logistic regression analysis were used to determine whether FBG and RBG were independent risk factors for in-hospital adverse events. MedCalc software was used to assess the predictive value of RBG and FBG in in-hospital adverse events and to assess predictive value difference between RBG and FBG. In all analyses, *P* < 0.05 was taken to indicate statistical significance.

## Results

### Comparison of baseline data, hematological parameters and coronary angiography data between elevated RBG group and control group

Our research found that of 958 patients with AMI who underwent emergency coronary angiography, more than one quarter of patients (265) had elevated RBG, 98 (36.98%) in them denied a history of diabetes.

There were significant differences in sex, age, hypertension, diabetes, previous MI, cerebral infarction, CKD, smoking, Killip class I, Killip class IV, BNP, WBC count, ALT, RBG, eGFR, FBG, TG, TC, HDL-C, LDL-C, LVEF, clopidogrel, ticagrelor, ACEI/ARBs, IABP, single-vessel lesion, double-vessel lesions and left main lesion between elevated RBG group and control group (*P* < 0.05). No significant statistical differences in the remaining indexes (Table 1).

**Table 1:** Comparison of baseline data, hematological parameters and coronary angiography data between elevated RBG group and control group

Variables	Elevated RBG group (*n* = 265)	Control group(*n* = 693)	*P* value
Gender(man)	193 (72.8%)	551 (79.5%)	0.026*
Age	64.08 ± 12.94	61.64 ± 13.47	0.019*
Height(cm)	166.71 ± 7.44	166.78 ± 7.54	0.92
Weight(kg)	68.55 ± 12.18	68.76 ± 11.38	0.844
BMI(kg/m^2^)	24.93 ± 3.87	24.44 ± 3.53	0.132
SBP(mmHg)	124.80 ± 24.80	126.52 ± 20.83	0.315
DBP(mmHg)	76.14 ± 16.29	76.98 ± 14.32	0.472
Hypertension	167 (63.0%)	384 (55.4%)	0.033*
Diabetes	174 (65.7%)	88 (12.7%)	< 0.001*
Previous MI	12 (4.5%)	10 (1.4%)	0.013*
Cerebral infarction	37 (14.0%)	65 (9.4%)	0.040*
CKD	30 (11.3%)	49 (7.1%)	0.032*
Smoking	119 (44.9%)	380 (54.8%)	0.006*
Killip grade			
I grade	158 (59.6%)	502 (72.4%)	< 0.001*
II grade	42 (15.8%)	112 (16.1%)	0.906
III grade	2 (0.8%)	9 (1.3%)	0.480
IV grade	63 (23.8%)	70 (10.1%)	< 0.001*
TNI P_50_(P_25_-P_75_)(ng/ml)	17.9 (3.2–25)	14 (3.53–25)	0.286
Myo P_50_(P_25_-P_75_) (ng/ml)	500 (241–900)	500 (239–900)	0.096
CK-MB P_50_(P_25_-P_75_)(ng/ml)	80 (26.9–292)	80 (44–303.25)	0.406
BNP P_50_(P_25_-P_75_) (pg/ml)	216.5 (55.9–862)	134 (43.3–393.25)	0.002*
WBC (*10^^^9/L)	12.46 ± 4.61	11.19 ± 3.68	< 0.001*
Hb (g/L)	133.21 ± 23.15	135.99 ± 20.71	0.098
PLT (*10^^^9/L)	207.48 (146.4–289.3)	204.68 (130.5–275.2)	0.564
RBG(mmol/L)	16.34 (13.2–20.6)	7.35 (6.8–9.6)	< 0.001*
ALT P_50_(P_25_-P_75_)IU/L)	53 (34–82)	44 (30–68.75)	0.001*
AST P_50_(P_25_-P_75_) (IU/L)	174.5 (79–323)	157.5 (76.75–262.25)	0.073
Scr P_50_(P_25_-P_75_) (μmol/L)	81 (71–105.5)	80.5 (69–92)	0.1
eGFR P_50_(P_25_-P_75_) (ml/min/1.73m^2^)	77.18 (54.95–96.24)	82.37 (68.89–97.27)	0.022*
UA(μmol/L)	364.18 (318.3–468.6)	366.75 (314.6–453.3)	0.079
FBG(mmoL/L)	12.00 ± 5.47	6.45 ± 2.02	< 0.001*
TG(mmoL/L)	2.12 (1.68–3.39)	1.67 (1.32–2.87)	0.004*
TC(mmoL/L)	4.27 (3.03–5.97)	4.48 (2.97–5.42)	0.044*
HDL-c(mmoL/L)	1.02 (0.82–1.88)	1.13 (1.01–1.96)	< 0.001*
LDL-c(mmoL/L)	2.62 (2.38–3.42)	2.80 (2.07–3.36)	0.017*
LVEF (%)	0.54 ± 0.12	0.58 ± 0.11	< 0.0010*
Aspirin	263 (99.2%)	681 (98.3%)	0.26
Clopidogrel	21 (7.9%)	95 (13.7%)	0.014*
Ticagrelor	244(%)	598 (86.3%)	0.014*
ACEI/ARB	112 (92.1%)	370 (53.4%)	0.002*
β-blocker	214 (80.8%)	580 (83.7%)	0.28
Statin	265 (100%)	688 (99.3%)	0.166
CCB	16 (6.0%)	44 (6.3%)	0.338
Furosemide	72 (27.1%)	177 (25.8%)	0.424
Antisterone	70 (26.4%)	179(%)	0.261
IABP	28 (10.6%)	35 (5.1%)	0.002*
CABG	5 (1.9%)	8 (1.2%)	0.381
Coronary lesions			
Single vessel lesion	44 (16.6%)	192 (27.7%)	< 0.001*
Double vessel lesions	95 (35.8%)	191 (27.6%)	0.012*
Triple vessel lesions	126 (47.5%)	310 (44.7%)	0.434
Number of stents			
Single stent	167 (63.0%)	415 (59.9%)	0.374
Two or more stents	44 (5.3%)	133 (19.2%)	0.356
Left main lesion	37 (14.0%)	62 (9.0%)	0.023*

*BMI* Body Mass Index, *SBP* Systolic blood pressure, *DBP* Diastolic blood pressure, *CKD* Chronic kidney diseases, *TNI* Troponin, *Myo* Myohemoglobin, *CK-MB* Creatine Kinase-MB, *BNP* Brain natriuretic peptide, *WBC* White blood cell, *Hb* Hemoglobin, *PLT* Platelet, *RBG* Random blood glucose, *ALT* Alanine transaminase, *AST* Aspartate transaminase, *Scr* Serum creatinine, *eGFR* Estimated glomerular filtration rate, *UA* Uric Acid, *FBG* Fasting blood glucose, *TG* Triglyceride, *TC* Total cholesterol, *HDL-c* High density lipoprotein-cholesterol, *LDL-c* Low density lipoproten-cholesterol, *LVEF* Left ventricular ejection fraction, *CCB* Calcium channel blocker, *IABP* Intra-aortic ballon pump, *CABG* Coronary Artery Bypass Grafting

### Comparison of baseline data, hematological parameters and coronary angiography data in elevated FBG group and normal FBG group

Over three-fifths of patients had elevated fasting blood glucose, and up to 347 (59.22%) were non-diabetic. There were significant differences in sex, BMI, diabetes, cerebral infarction, smoking, Killip class I, Killip class IV, TNI, Myo, BNP, WBC, RBG, ALT, AST, FBG, HDL-C, LVEF, clopidogrel, ticagrelor, ACEI/ARB, furosemide, antisterone, IABP, single-vessel disease, triple-vessel disease, number of stents and left main lesion between the elevated FBG group and the normal FBG group (*P* < 0.05) (Table 2).

**Table 2:** Comparison of baseline data, hematological parameters and coronary angiography data in elevated FBG group and control group

Variables	Elevated FBG group (*n* = 589)	Normal FBG group(*n* = 369)	*P* value
Gender(man)	433 (73.5%)	303 (82.1%)	0.002*
Age	62.91 ± 13.01	61.72 ± 13.84	0.185
Height(cm)	166.77 ± 7.64	166.70 ± 7.20	0.087
Weight(kg)	69.02 ± 12.20	68.15 ± 11.73	0.319
BMI(kg/m^2^)	24.77 ± 3.70	24.09 ± 3.71	0.012*
SBP(mmHg)	127.08 ± 23.18	125.34 ± 19.14	0.235
DBP(mmHg)	76.89 ± 15.05	76.44 ± 13.84	0.467
Hypertension	349 (59.3%)	202 (54.7%)	0.169
Diabetes	242 (41.1%)	20 (5.4%)	< 0.001*
Previous MI	15 (2.5%)	7 (1.9%)	0.514
Cerebral infarction,	86 (14.6%)	16 (4.3%)	< 0.001*
CKD	54 (9.2%)	25 (6.8%)	0.190
Smoking	283 (48.0%)	216 (58.5%)	0.002*
Killip grade			
I grade	367 (62.3%)	293 (79.4%)	< 0.001*
II grade	103 (17.5%)	51 (13.8%)	0.133
III grade	9 (1.5%)	2 (0.5%)	0.163
IV grade	99 (16.8%)	34 (9.2%)	< 0.001*
TNI P_50_(P_25_-P_75_)(ng/ml)	17 (3.98–25)	11 (2.3–25)	0.002*
Myo P_50_(P_25_-P_75_) (ng/ml)	500 (219.75–900)	500 (206.25–900)	0.016*
CK-MB P_50_(P_25_-P_75_)(ng/ml)	80 (36.45–297)	80 (37.5–246.5)	0.260
BNP P_50_(P_25_-P_75_) (pg/ml)	173 (49.75–587.5)	112 (41.45–347.5)	0.001*
WBC (*10^^^9/L)	12.29 ± 4.45	10.47 ± 2.94	< 0.001*
Hb (g/L)	134.5 ± 23.11	135.99 ± 20.71	0.066
PLT (*10^^^9/L)	209.87 (160.3–268.3)	201.7 (170.4–259.2)	0.051
RBG(mmol/L)	11.25 (9.3–16.5)	7.22 (6.8–10.0)	< 0.001*
ALT P_50_(P_25_-P_75_)IU/L)	48 (33–79)	39 (29–59)	< 0.001*
AST P_50_(P_25_-P_75_) (IU/L)	145 (60–219)	171 (89–309)	< 0.001*
Scr P_50_(P_25_-P_75_) (μmol/L)	81 (67–98)	80 (72–91)	0.862
eGFR P_50_(P_25_-P_75_) (ml/min/1.73m^2^)	80.9 (62.22–97.88)	81.7 (69.2–96.2)	0.704
UA(μmol/L)	376.41 (280.3–390.3.2)	365.35 (249.5–378.4)	0.152
FBG(mmoL/L)	9.79 ± 2.67	5.22 ± 0.52	< 0.001*
TG(mmoL/L)	1.83 (1.28–2.56)	1.64 (0.89–2.06)	0.051
TC(mmoL/L)	4.46 (4.13–5.84)	4.42 (4.02–5.73)	0.667
HDL-c(mmoL/L)	1.12 (0.83–1.46)	1.07 (0.95–1.47)	0.046*
LDL-c(mmoL/L)	2.77 (2.19–3.07)	2.80 (2.26–3.26)	0.682
LVEF (%)	0.53 ± 0.38	0.68 ± 0.34	< 0.001*
Aspirin	581 (98.6%)	364 (98.6%)	0.997
Clopidogrel	61 (10.4%)	55 (14.9%)	0.028*
Ticagrelor	528 (89.6%)	314 (85.1%)	0.028*
ACEI/ARB	277 (47.0%)	205 (55.6%)	0.01*
β-blocker	494 (83.9%)	300 (81.3%)	0.304
Statin	586 (99.5%)	367 (99.5%)	0.946
CCB	30 (5.1%)	30 (8.1%)	0.059
Furosemide	174 (29.5%)	75 (20.3%)	0.002*
Antisterone	172 (29.2%)	77 (20.9%)	0.004*
IABP	51 (8.7%)	12 (3.3%)	0.001*
CABG	10 (17.1%)	3 (0.8%)	0.249
Coronary lesions			
Single vessel lesion	101 (29.0%)	135 (36.6%)	< 0.001*
Double vessel lesions	171(%)	115 (31.2%)	0.483
Triple vessel lesions	284 (48.2%)	152 (41.2%)	0.034*
Number of stents			
Single stent	340 (57.7%)	242 (65.6%)	0.015*
Two or more stents	124 (21.1%)	53 (14.4%)	0.009*
Left main lesion	75 (12.7%)	24 (6.5%)	0.002*

### The incidence of in-hospital adverse events in elevated RBG patients, elevated FBG patients and control group

In the elevated RBG group, 56 patients died, 6 patients had nonfatal recurrent MI, 6 patients underwent target vessel revascularization, 1 patient had a stroke, 9 patients had hemorrhage, 57 patients suffered cardiogenic shock, and 25 patients had Malignant arrhythmia. Chi-square analysis showed that the incidence of death, cardiogenic shock, malignant arrhythmia and hemorrhage in elevated RBG group were all significantly higher than that in control group (*P* < 0.001, *P* = 0.014, P < 0.001, P < 0.001). In the FBG-elevated group, 72 patients died, 7 patients had non-fatal recurrent MI, 7 patients had target vessel revascularization, 1 patient had a stroke, 20 had hemorrhage, and 89 patients suffered cardiogenic shock, 32 patients developed malignant arrhythmia. Chi-square analysis showed that the incidence of death, cardiogenic shock, malignant arrhythmia and hemorrhage significantly increased in FBG elevated group than in normal FBG group (*P* < 0.001, *P* = 0.013, P < 0.001, *P* = 0.003). The incidence of non-fatal recurrent myocardial infarction, target vessel revascularization and stroke were relatively low in both elevated RBG and elevated FBG group, no statistical difference were found between elevated RBG, elevated FBG group and control group (Table 3).

**Table 3:** Comparison of incidence of in-hospital adverse events in patients with elevated RBG, elevated FBG and control group

Variables	Elevated RBG group	Normal RBG group	*P* value	Elevated FBG group	Normal FBG group	*P* value
Death	56 (265)	24 (693)	< 0.001*	72 (589)	5 (369)	< 0.001*
Nonfatal recurrent myocardial infarction	6 (265)	4 (693)	0.052	7 (589)	3 (369)	0.818
Stroke	1 (265)	0 (693)	0.617	1 (589)	0 (369)	1
Hemorrhage	9 (265)	14 (693)	0.014*	20 (589)	3 (369)	0.013*
Cardiac shock	57 (265)	49 (693)	< 0.001*	89 (589)	17 (369)	< 0.001*
Malignant arrhythmia	25 (265)	13 (693)	< 0.001*	32 (589)	6 (369)	0.003*

* *P* < 0.05;

### Binary logistic regression analysis for in-hospital adverse events

Binary logistic regression showed that age, hypertension, diabetes, FBG and RBG were all independent risk factors for in-hospital adverse events in ST-segment elevation myocardial infarction patients (*P* = 0.037, *P* = 0.039, *P* = 0.043, P = 0.043, *P* = 0.048, respectively; the corresponding 95% CI = 1.39–2.07,1.15-1.67,1.79-3.85,1.12-1.98,1.04–1.63, respectively) (Table 4).

**Table 4:** Binary logistic regression analysis for in-hospital adverse events

Variables	OR	95% CI	*P* value
Gender	1.17	0.93–1.95	0.075
Age	1.63	1.39–2.07	0.037*
Hypertension	1.34	1.15–1.67	0.039*
Diabetes	2.47	1.79–3.85	0.043*
Smoking	2.453	0.75–3.49	0.785
FBG	1.740	1.12–1.98	0.043*
RBG	1.205	1.04–1.63	0.048*

*RBG* Random blood glucose, *FBG* Fasting blood glucose

### ROC curve

**Fig. 1:**
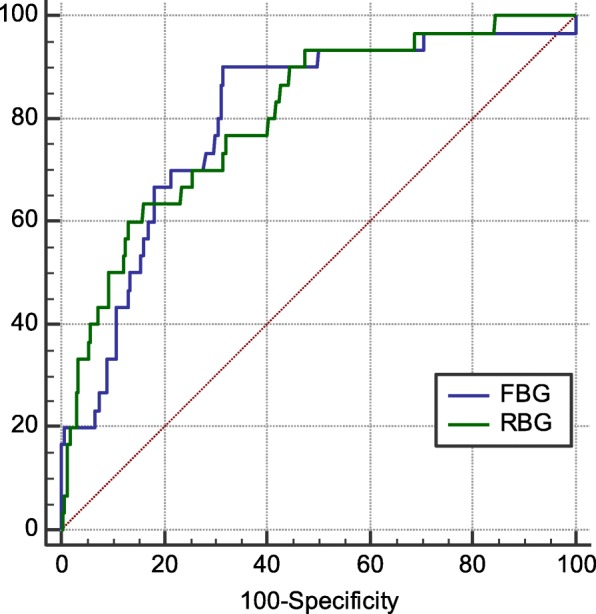
The ROC cure of RBG and FBG for predicting in-hospital death

The AUC and 95% CI of RBG and FBG for predicting in-hospital death in AMI patients were 0.789, 0.759~0.816, (*P* < 0.0001); 0.810, 0.783~0.835 (P < 0.0001) respectively. The cut-off values of RBG and FBG were 13.82 and 7.35 mmol/L, respectively, the corresponding sensitivity and specificity were 61.11, 86.44%; 90.62, 68.05%, respectively. There was no significant difference in predicting in-hospital death between them (*P* = 0.462) (Fig. 1).

**Fig. 2:**
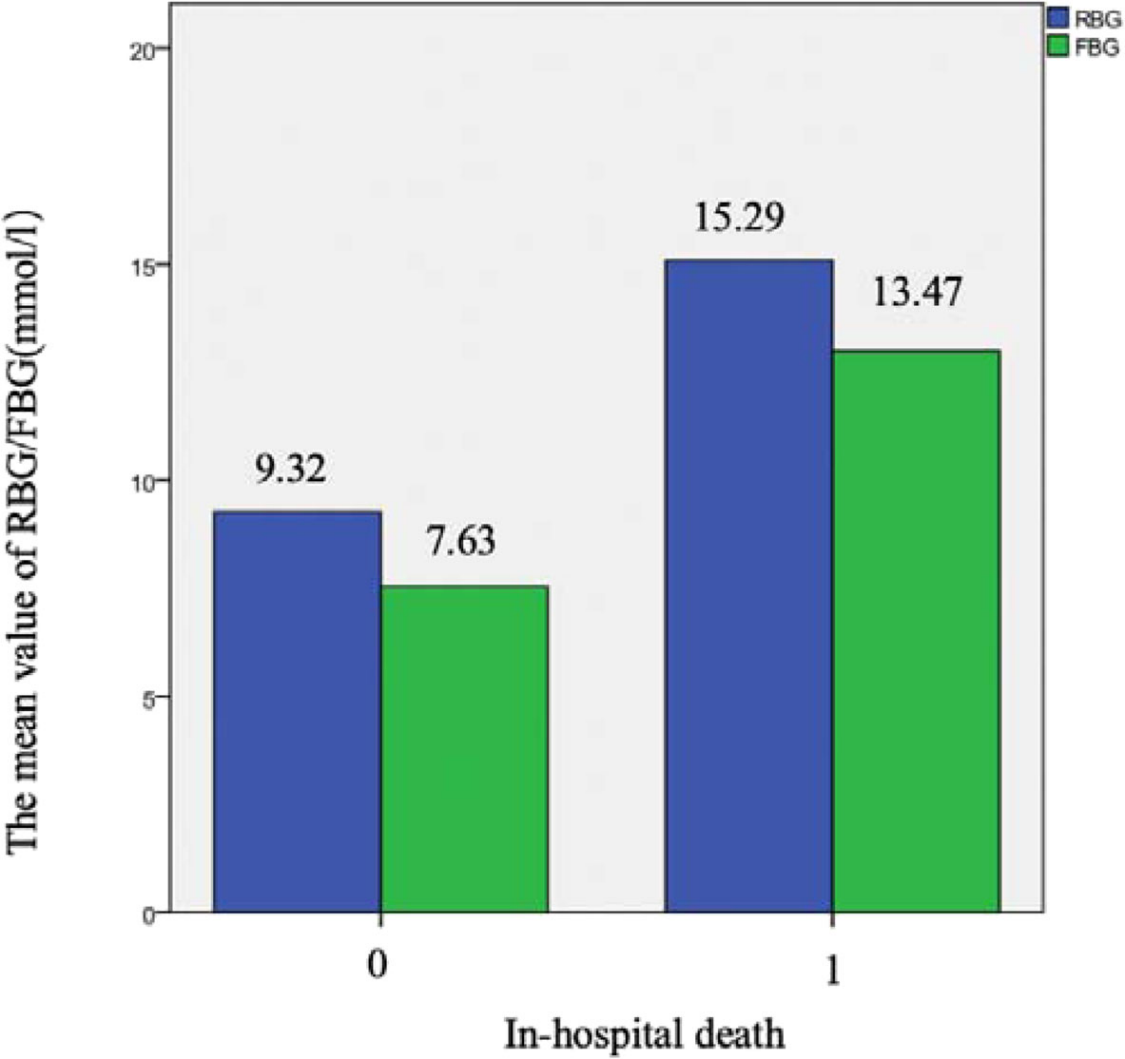
The mean of RBG and FBG in in-hospital death and control group

The average RBG and FBG in AMI patients who died in hospital were 15.29 and 13.47 mmol/L, respectively. The average RBG and RBG levels in normal RBG and normal FBG group were 9.32 and 7.63 mmol/L, respectively (Fig. 2).

**Fig. 3:**
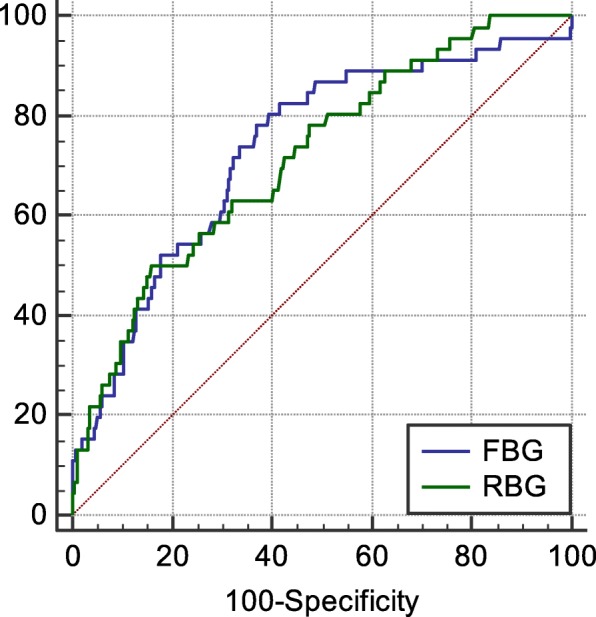
The ROC cure of RBG and FBG for predicting cardiac shock

The AUC and 95%CI of RBG and FBG for predicting cardiac shock were 0.703、0.670–0.734 (*P* < 0.0001); 0.746, 0.717–0.771 (*P* < 0.0001) respectively, the cut-off value were 13.14,6.96 mmol/L respectively, and the corresponding sensitivity and specificity were 50.0, 83.43%; 79.59, 62.68%. There was no statistical difference in predicting cardiac shock of RBG and FBG (*P* = 0.5704) (Fig. 3).

**Fig. 4:**
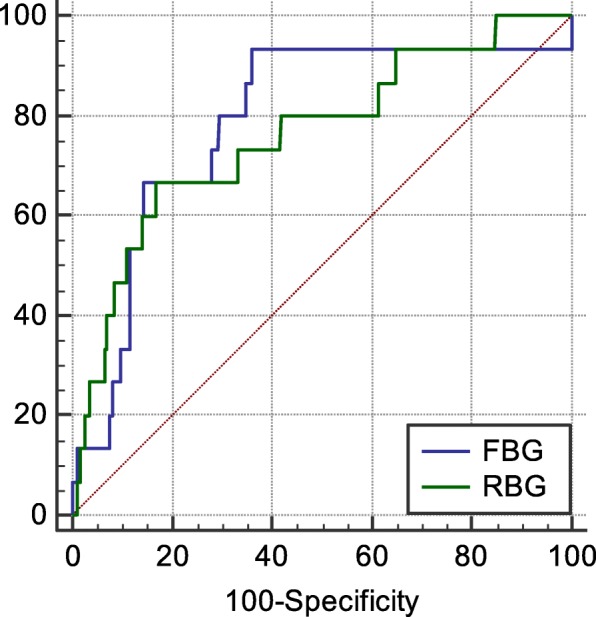
The ROC curve of RBG and FBG for predicting malignant arrhythmia

The AUC and 95% CI of RBG and FBG for predicting malignant arrhythmia were 0.740, 0.709–0.770 (*P* < 0.0001); 0.798, 0.771–0.824 (P < 0.0001) respectively, the cut-off value were 13.28, 7.19 mmol/L respectively, and the corresponding sensitivity and specificity were 66.7%、82.4%; 93.7%、63.6%. There was no statistical difference in predicting malignant arrhythmia of RBG and FBG (*P* = 0.6540) (Fig. 4).

### Subgroup analysis for nondiabetic patients

**Fig. 5:**
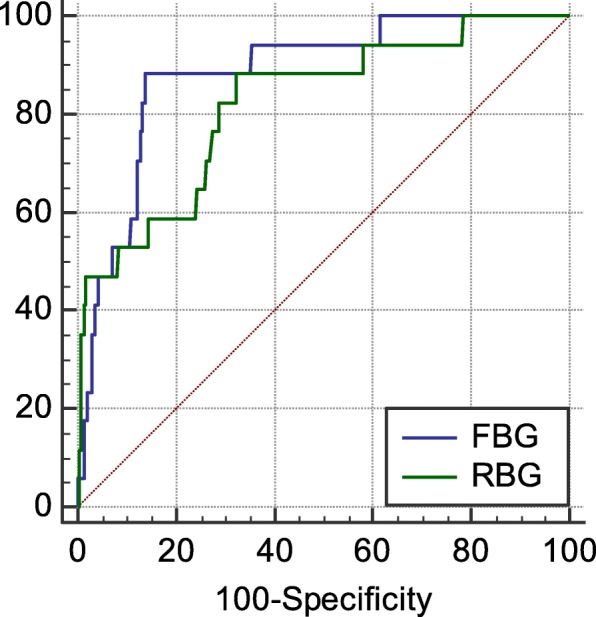
The ROC curve of RBG and FBG for predicting death in nondiabetic patients

In nondiabetic AMI patients, the AUC and 95%CI of RBG and FBG for predicting in-hospital death were 0.808、0.774–0.839 (*P* < 0.0001); 0.891、0.865–0.914 (P < 0.0001) respectively, the cut-off value were 8.00, 7.35 mmol/L respectively, and the corresponding sensitivity and specificity were 82.6, 67.0%; 88.89, 86.35%. There was no statistical difference in predicting in-hospital death of RBG and FBG (*P* = 0.762) (Fig. 5).

## Discussion

Our research showed that the incidences of elevated RBG and elevated fasting blood glucose were significantly high, in which non-diabetics accounted for relatively high proportion. Elevated RBG and elevated FBG group have significantly higher incidence of in-hospital death, malignant arrhythmia, hemorrhage and cardiac shock compared with normal glucose patients. The incidence of nonfatal recurrent MI、target vessel revascularization and stroke were all extremely low, hence, no significance was found in elevated glucose and normal glucose group. ROC curve indicated that FBG and RBG had outstanding predictive value in in-hospital death, hemorrhage, cardiogenic shock and malignant arrhythmia during hospitalization. The sensitivity and specificity can be as high as 90.62, and 68.05% when predicting the possibility of the in-hospital death AMI patient’s FBG over 7.35 mmol/L. The AUC area of ​​the FBG predicting adverse events during hospitalization was significantly higher than that of the RBG, but no statistically significant difference was found between them in terms of predictive value. A multicenter large sample study is needed to further clarify the difference in predictive value on poor prognosis. The ROC curve in subgroup analysis showed that RBG and FBG have higher predictive value for in-hospital death in non-diabetic patients with AMI. When FBG is 7.35 mmol/L, the corresponding sensitivity and specificity are as high as 88.89 and 86.35%. Vergès B and his colleagues underscores the high prevalence of IFG (25%) and highlighted the clinical relevance of 6.1 mmol/l, as a cutoff value to define short-term outcome in AMI patients [17]. Random blood glucose and fasting blood glucose are both clinically commonly used and easily available indicators. Our research showed that FBG, along with RBG, has certain clinical value on risk stratification and prognosis evaluation in patients with AMI. In addition, we also provide random blood glucose and fasting blood glucose thresholds that can be used to predict adverse events in hospitals including death, which is of significant use in clinical practice.

Acute hyperglycemia refers to the transient high blood glucose that occurs during the development of disease. There is no consensus on the appropriate definition of hyperglycemia for patients with AMI, blood glucose of 10.0 mmol/L or 11.0 mmol/L on admission is used most frequently to define acute hyperglycemia. Elevated RBG belongs to hyperglycemia, the prevalence of elevated RBG in AMI varies a lot from study to study according to its definition [5]. In general, about half of patients with STEMI have elevated RBG [18]. Plenty of studies have demonstrated that patients with AMI and elevated RBG on admission have high incidence of mortality, low left ventricular ejection fraction and short-term poor prognosis in AMI patients [19]. Tamer M. Moustafa discovered that elevated admission blood glucose level was an independent risk factor in prediction of cardiac events and mortality, admission glucose was closely associated with poor adverse outcome and more severe multi-vessel coronary lesions in STEMI patients without diabetes. They proposed that the cut-off value of admission glucose was 160 mg/dl [8]. Reviewing 141,680 patients aged ≥65 years with AMI, the Cooperative Cardiovascular Project revealed that the 30-day and 1-year mortality rates were linearly associated with admission blood glucose level [20]. JACSS Investigators enrolled 3750 AMI patients, demonstrating a linear relation between admission blood glucose level and in-hospital mortality in nondiabetic patients [21]. In a meta-analysis of 1856 AMI patients (5), the relative risk of in-hospital death in non-diabetic patients with elevated RBG was 3.9 times higher than patients with normal blood glucose (95% CI = 2.9–5.4), diabetic patients combined with admission elevated RBG had 1.7 times higher risk of death during hospitalization than that in normal population (95% CI =1.2–2.4) [5]. Although it is well known that elevated admission blood glucose level is closely associated with poor prognosis in AMI patients, studies have shown that persistent hyperglycemia during acute myocardial infarction has a better predictive value for in-hospital mortality than admission glucose. Considering the simplicity of clinical implementation and calculation, the average blood glucose level seems to be the best method for assessing the prognosis of AMI patients with hyperglycemia. The J-shaped curve of mean blood glucose level and in-hospital mortality showed that not only hyperglycemia, hypoglycemia can also increase mortality in AMI patients [7]. Studies need to further clarify the relationship between hyperglycemia in AMI and poor prognosis.

Elevated fasting glucose was also associated with the risk of cardiovascular disease [22]. With the increasement of blood glucose level, even below the current diagnosis criteria for diabetes, the risk of developing cardiovascular disease increases [23]. The National Hospital of Singapore conducted a five-year follow-up of 2295 patients with newly diagnosed IFG, finding that 492 (21.4%) subjects developed T2DM within 5 years, and 20 (0.9%) developed AMI [24]. A retrospective multicenter 5-year cohort study performed on 9161 Chinese IFG subjects attending 23 general outpatient clinics showed 1998 participants eventually developed T2DM. The 5-year cumulative incidence was 0.218 [25]. A prospective study enrolling 2733 consecutive AMI subjects underwent percutaneous coronary intervention (PCI)indicated impaired glucose tolerance bearing similar long-term prognosis as diabetes [26]. These researches showed the association between elevated fasting blood glucose and diabetes, elevated fasting blood glucose significantly increases the incidence of suffering AMI. Therefore, we must pay attention to elevated glucose, even not yet reached the diagnostic criteria for diabetes.

Mechanically speaking, hyperglycemia has a direct detrimental effect on ischemic myocardium through several mechanisms, including oxidative stress, inflammation, apoptosis, endothelial dysfunction, hypercoagulation, platelet aggregation and impairment of ischemic preconditioning. Specifically, acute hyperglycemia exaggerates inflammation by the oxidative mechanism [27]. Animal experiments show that SGLT2 inhibitors can reduce the mortality of myocardial infarction in diabetic mice through cardiac energy metabolism and protective modification of antioxidant proteins, suggesting that hyperglycemia is associated with energy metabolism disorder and oxidative stress in myocardial infarction [28]. Risso reported that intermittent high glucose enhanced apoptosis of human endothelial cells [29]. Besides, acute hyperglycemia can lead to endothelial dysfunction, hyperglycemia also affects coronary collateral blood flow [30]. Hyperglycemia also stimulates blood clotting and platelet aggregation, which can upregulate coagulation activation markers including thrombin antithrombin complex and soluble tissue factor [31].

In addition to RBG and FBG, there are some other studies focusing on the relationship between blood glucose and AMI. Considering that the risk of adverse events such as death and recurrent angina in AMI patients with diabetes is quite different, Suzanne V. Arnold, et al. developed a prediction model for long-term mortality and angina to stratify AMI patients with diabetes [9]. Considering the change in FBG concentrations over time, Cheng Jin conducted a prospective cohort study included 68,297 non-diabetic participants, they found that discrete FBG trajectories were significantly related to subsequent risk of MI. These observations suggest that trajectories of FBG may be significant for predicting MI and other cardiovascular risk [32]. Su G also demonstrated that elevated admission glycemic variability appears more important than admission glucose in predicting 1-year MACE in AMI patients [33]. In addition, postprandial hyperglycemia also acts as an accelerating factor in atherosclerosis and other cardiovascular disease [34].

When it comes to the treatment for hyperglycemia, recent randomized controlled trial of DPP-4 inhibitors in T2DM patients after acute coronary syndrome didn’t show beneficial effects on cardiovascular outcomes [35]. Continuous insulin infusion is recommended as first-line treatment for AMI patients combined with acute hyperglycemia, taking account of the safety and efficacy, guidelines recommend taking insulin-based regimen to achieve and maintain glucose levels < 10.0 mmol/l, and emphasize avoiding hypoglycemia [36].

## Conclusion

Our study demonstrates that elevated RBG and elevated FBG are very common in AMI patients, among them many patients non-diabetic. Both RBG and FBG are closely related to poor in-hospital prognosis. RBG and FBG have significant predictive value in adverse events during hospitalization, besides, RBG and FBG share similar predictive value on in-hospital adverse events. Finally, we provide the threshold for RBG and FBG to predict the adverse events in hospital, which has certain value in clinical work. This research still has the following shortcomings: Firstly, this study is a single-center retrospective and observational design, large-scale multi-center prospective RCT design needs to be carried out to further evaluate the predictive value of RBG, FBG on in-hospital poor prognosis; Secondly, Long-term follow-up is requisite to evaluate long-term adverse effects of RBG and FBG and blood glucose fluctuations.

## Data Availability

The data that support the findings of this study are available from Zhongda Hospital, the data are available from the authors upon reasonable request and with permission from Zhongda Hospital.

## Abbreviations

AMI: Acute myocardial infarction
AUC: Area under curve
CABG: Coronary artery bypass grafting
FBG: Fasting blood glucose
IABP: Intra-aortic balloon pump
MACCE: Major adverse cardiovascular and cerebrovascular events
PCI: Percutaneous coronary intervention
RBG: Random blood glucose
STEMI: ST-segment elevation acute myocardial infarction
T2DM: Type 2 diabetes mellitus
